# Analysis of Fractional Dynamic Systems

**DOI:** 10.1155/2014/760634

**Published:** 2014-04-23

**Authors:** Fawang Liu, Richard Magin, Changpin Li, Alla Sikorskii, Santos Bravo Yuste

**Affiliations:** ^1^School of Mathematical Sciences, Queensland University of Technology, P.O. Box 2434, Brisbane, QLD 4001, Australia; ^2^Department of Bioengineering, University of Illinois, 851 South Morgan Street, Chicago, IL 60607, USA; ^3^Department of Mathematics, Shanghai University, Shanghai 200444, China; ^4^Department of Statistics and Probability, Michigan State University, East Lansing, MI 48824, USA; ^5^Departamento de Física, Universidad de Extremadura, Avenida de Elvas s/n, 06071 Badajoz, Spain

Due to the extensive applications of fractional differential equations (FDEs) in engineering and science, research in this area has grown significantly. Fractional Dynamic Systems are described by FDEs, and this special issue consists of 8 original articles covering various aspects of FDEs and their applications written by prominent researchers in the field.

Paper titled “*Sinc-Chebyshev collocation method for a class of fractional diffusion-wave equations*” by Z. Mao et al. investigates the numerical solution for a class of fractional diffusion-wave equations with a variable coefficient. The approach is based on the collocation technique where the shifted Chebyshev polynomials in time and the sinc functions in space are utilized, respectively.

Paper titled “*Leapfrog/finite element method of fractional diffusion equation*” by Z. Zhao and Y. Zheng analyzes a fully discrete leapfrog/Galerkin finite element method for the numerical solution of the space fractional order diffusion. The fractional diffusion equations are discretized in space by the finite element method and in time by the explicit leap-frog scheme. For the resulting fully discrete, conditionally stable scheme the authors use an L2-error bound of finite element accuracy and of second order in time.

Paper titled “*Determination of coefficients of high-order schemes for Riemann-Liouville derivative*” by R. Wu et al. studies and develops recursion formulas to compute the coefficients in the higher-order schemes approximating Riemann-Liouville integrals and derivatives. The fractional Runge's phenomena are observed when the* p*th-order numerical scheme (*p *= 7, 8, 9, 10) is used, which means that* p*th-order algorithms (*p* ≥ 7) for Riemann-Liouville derivative seem not to be appropriate.

Paper titled “*Numerical solution of some types of fractional optimal control problems*” by N. H. Sweilam et al. develops two different approaches based on the spectral method for some types of fractional optimal control problems. The spectral method given here is based on the Chebyshev polynomials to approximate the unknown functions. Necessary and sufficient optimality conditions are obtained in the first approach, where the Hamiltonian functional is defined. In the second approach the Clenshaw and Curtis procedure for the numerical integration of nonsingular functions and Rayleigh-Ritz method are used to evaluate both the state and control variables.

Paper titled “Stability analysis of distributed order fractional Chen system” by H. Aminikhah et al. derives the sufficient and necessary conditions of stability of nonlinear distributed order fractional system and then the integer-order Chen system is generalized into the distributed order fractional domain. Based on the asymptotic stability theory of nonlinear distributed order fractional systems, the stability of distributed order fractional Chen system is discussed.

Paper titled “*Monotonicity, concavity, and convexity of fractional derivative of functions*” by X.-F. Zhou et al. discusses the monotonicity, the concavity, and the convexity of some functions arising in solutions of fractional differential equations. The results can be used in describing properties of the solutions.

Paper titled “*A directed continuous time random walk model with jump length depending on waiting time*” by L. Shi et al. proposes a coupled directed continuous time random walk model, where the random walker jumps toward one direction and the waiting time between jumps affects the subsequent jump. The limit distribution of the continuous time random walk and the corresponding evolving equations are derived.

Finally, paper titled “*A parallel algorithm for the two-dimensional time fractional diffusion equation with implicit difference method*” by C. Gong et al. introduces and discusses a parallel algorithm for a two-dimensional time fractional differential equation (2D-TFDE). A task distribution model with virtual boundary is designed for this parallel algorithm.

Thus, this special issue provides a wide spectrum of current research in the area of analysis of Fractional Dynamic Systems, and we hope that experts in this and related fields find it useful.

*Fawang Liu* *Fawang Liu* *Richard Magin* *Richard Magin* *Changpin Li* *Changpin Li* *Alla Sikorskii* *Alla Sikorskii* *Santos Bravo Yuste* *Santos Bravo Yuste*

